# Supplementary material for: Early Bronchoscopy Improves Extubation Rates after Out-of-Hospital Cardiac Arrest: A Retrospective Cohort Analysis
Source: J Clin Med. 2021 Jul 9;10(14):3055. doi: 10.3390/jcm10143055 (PMC8306153; doi:10.3390/jcm10143055)
Supplement: Supplementary file 1 [file jcm-10-03055-s001.zip › jcm-1280383-supplementary.pdf]

Supplementary Material.

Table S1. Baseline Clinical and Demographic Characteristics of 111 patients after OHCA and early or late bronchoscopy

	**All patients with Bronchoscopy** *n* **(%)**	**Early Bronchoscopy (< 48hrs)** *n* **(%)**	**Late Bronchoscopy (> 48hrs)** *n* **(%)**	***p*-value**
Patients	111	91	20	
Female	31 (27.9)	21 (23.1)	10 (50.0)	0.015
Age (yrs.)	63.7 ± 15.4	62.7 ± 15.7	67.9 ± 13.6	0.179
Ongoing CPR on admission	12 (10.8)	9 (9.9)	3 (15.0)	0.450
Shockable rhythm	74 (66.6)	59 (64.8)	15 (75.0)	0.383
Cause of OHCA				
Myocardial ischemia	66 (59.5)	52 (57.1)	14 (70.0)	0.289
Pulmonary embolism	6 (5.4)	5 (5.5)	1 (5.0)	1.000
Rhythm event	13 (14.3)	12 (13.2)	1 (5.0)	0.457
Unknown/other	26 (23.4)	22 (24.2)	4 (20.0)	0.779
Time to ROSC (min.)	18.9 ± 15.9	17.8 ± 14.7	25.8 ± 22.2	0.166
CVRF				
Arterial hypertension	60 (54.1)	50 (55.0)	10 (50.0)	0.688
Hyperlipoproteinemia	42 (37.8)	34 (37.4)	8 (40.0)	0.826
Active nicotine abuse	41 (37.0)	36 (40.0)	5 (25.0)	0.222
Pre-existing heart failure	21 (19.0)	20 (22.0)	1 (5.0)	0.115
Pre-existing heart rhythm disorder (e.g. atrial fibrillation)	25 (22.5)	17 (18.7)	8 (40.0)	0.039
Pre-existing pulmonary disease (e.g. COPD)	19 (17.1)	16 (17.6)	3 (15.0)	1.000
Diabetes type 2	20 (18.0)	15 (16.5)	5 (25.0)	0.370
Family history of SCD	9 (8.1)	8 (8.8)	1 (5.0)	1.000
Laboratory measurements				
Lactate (mmol/l) day 1	6.81 ± 6.06	6.65 ± 6.06	7.64 ± 6.25	0.608
Lactate (mmol/l) day 3	2.80 ± 3.10	2.84 ± 3.32	2.59 ± 1.64	0.780
Lactate (mmol/l) day 5	1.50 ± 1.41	1.56 ± 1.56	1.26 ± 0.45	0.437
Lactate (mmol/l) day 7	1.45 ± 1.40	1.51 ± 1.57	1.22 ± 0.52	0.499
CRP (mg/l) day 1	21.2 ± 44.9	21.6 ± 47.3	19.5 ± 34.3	0.851
CRP (mg/l) day 3	163.4 ± 84.2	162.7 ± 87.2	166.8 ± 70.4	0.855
CRP (mg/l) day 5	157.4 ± 95.2	147.0 ± 91.8	198.5 ± 99.8	0.034
CRP (mg/l) day 7	121.8 ± 165.4	109.2 ± 180.0	160.1 ± 100.3	0.247
Catecholamine use day 1	110 (99.1)	90 (98.9)	20 (100.0)	1.000
Catecholamine use day 3	103 (92.8)	83 (92.2)	20 (100.0)	0.582
Catecholamine use day 5	65 (58.6)	48 (52.7)	17 (85.0)	0.008
Catecholamine use day 7	40 (36.0)	26 (28.6)	14 (70.0)	0.003
Ventilation parameters				
PEEP (mbar) day 1	6.23 ± 1.62	6.29 ± 1.67	5.97 ± 1.39	0.459
PEEP (mbar) day 3	6.63 ± 2.43	6.58 ± 2.36	6.85 ± 2.79	0.669
PEEP (mbar) day 5	6.64 ± 2.50	6.50 ± 2.40	7.10 ± 2.83	0.402
PEEP (mbar) day 7	6.39 ± 2.36	6.45 ± 2.45	6.21 ± 2.20	0.743
pO_2/FiO_2 day 1	295.7 ± 124.2	297.2 ± 121.1	288.2 ± 141.8	0.781
pO_2/FiO_2 day 3	273.8 ± 100.6	272.4 ± 106.4	279.8 ± 70.75	0.774
pO_2/FiO_2 day 5	292.4 ± 99.10	290.7 ± 102.4	299.8 ± 85.96	0.729
pO_2/FiO_2 day 7	276.9 ± 106.9	278.5 ± 112.9	271.7 ± 87.86	0.822
